# Health-related quality of life in patients with COVID-19; international development of a patient-reported outcome measure

**DOI:** 10.1186/s41687-022-00434-1

**Published:** 2022-03-26

**Authors:** Cecilie Delphin Amdal, Katherine Taylor, Dagmara Kuliś, Ragnhild Sørum Falk, Andrew Bottomley, Juan Ignacio Arraras, James Harold Barte, Anne Sophie Darlington, Kristin Hofsø, Bernard Holzner, Nina Marie Høyning Jørgensen, Melissa Paulita Mariano, Madeline Pe, Claire Piccinin, Nicola Riccetti, Melanie Schranz, Sally Wheelwright, Kristin Bjordal

**Affiliations:** 1 Research Support Services, Oslo University Hospital, Sogn Arena, Nydalen, Post Box 4950, 0424 Oslo, Norway; 2 Department of Oncology, Oslo University Hospital, Oslo, Norway; 3 Institute of Medical Biostatistics, Epidemiology and Informatics (IMBEI), University Medical Centre of Johannes Gutenberg University, Mainz, Germany; 4 Quality of Life Department, EORTC, Brussels, Belgium; 5 Servicio de Navarro de Salud, Pamplona, Spain; 6 University of the East Ramon Magsaysay Memorial Medical Center, Quezon City, Philippines; 7 School of Health Sciences, University of Southampton, Southampton, UK; 8 Division of Emergencies and Critical Care, Oslo University Hospital, Oslo, Norway; 9 Lovisenberg Diaconal University College, Oslo, Norway; 10 Innsbruck Medical University, Innsbruck, Austria; 11 Medical Library at Oslo University Hospital, University of Oslo Library, Oslo, Norway; 12 Azienda Unità Sanitaria Locale (USL) Umbria N.2, Terni, Italy; 13 Health Sciences, University of Southampton, Southampton, UK; 14 Faculty of Medicine, University of Oslo, Oslo, Norway

**Keywords:** COVID-19, Quality of life, Symptoms, Patient-reported outcome measure, Questionnaire, PROM

## Abstract

**Background:**

We aimed to create a questionnaire to assess the health-related quality of life including functioning, symptoms, and general health status of adult patients with current or previous COVID-19. Here, we report on Phase I and II of the development.

**Methods:**

Internationally recognized methodology for questionnaire development was followed. In Phase I, a comprehensive literature review was performed to identify relevant COVID-19 issues. Decisions for inclusion, exclusion, and data extraction were completed independently in teams of two and then compared. The resulting issues were discussed with health care professionals (HCPs) and current and former COVID-19 patients. The input of HCPs and patients was carefully considered, and the list of issues updated. In Phase II, this updated list was operationalized into items/questions.

**Results:**

The literature review yielded 3342 publications, 339 of which were selected for full-text review, and 75 issues were identified. Discussions with 44 HCPs from seven countries and 52 patients from six countries showed that psychological symptoms, worries, and reduced functioning lasted the longest for patients, and there were considerable discrepancies between HCPs and patients concerning the importance of some of the symptoms. The final list included 73 issues, which were operationalized into an 80-item questionnaire.

**Conclusion:**

The resulting COVID-19 questionnaire covers health–related quality of life issues relevant to COVID-19 patients and is available in several languages. The next steps include testing of the applicability and patients’ acceptability of the questionnaire (Phase IIIA) and preliminary psychometric testing (Phase IIIB).

**Supplementary Information:**

The online version contains supplementary material available at 10.1186/s41687-022-00434-1.

## Background

The coronavirus disease (COVID-19) carries with it a complex symptom burden in the acute and sub-acute phase and recent evidence also indicates the presence of long-term side effects and reduced health-related quality of life (HRQoL) [1–4]. HRQoL is defined as a multidimensional concept that includes domains related to physical, mental, emotional and social functioning [5], and thereby include patient-reported symptoms. The societal implications of the pandemic, such as quarantine and social distancing, may also have negative impacts on patients’ HRQoL. The immense burden on healthcare systems has led to less individualized care [6] especially as healthcare professionals (HCPs) struggle to keep up with emerging evidence, often with limited resources at their disposal. Initially, identified symptoms were dry cough, shortness of breath, fever, muscle pain, and fatigue [7, 8], but additional symptoms and functional deficits have increasingly been reported, such as skin rash [9], smell and taste disturbances, facial pain, nasal obstruction [10], and neurological manifestations [11]. Elderly patients with COVID-19 may present with atypical symptoms, such as confusion, loss of appetite, and dizziness [12]. In addition, treatments being tested in clinical trials may have serious side effects [13, 14].

At the onset of the pandemic, appropriate, validated COVID-19 specific patient-reported outcome measures (PROM) were not available and researchers were limited to using symptom checklists and generic HRQoL-questionnaires [2, 15], which fail to capture the full spectrum of relevant issues. The PROMIS Global Health survey and the PROMIS Dyspnea Functional Limitations survey [16] was used in one study, while another study used an iterative peer review process to develop an assessment tool of post-discharge symptoms and rehabilitation needs [2]. These approaches lack the critical aspects of securing content validity of the assessment by reviewing the literature and interviewing patients for relevant issues.

Lifestyle changes and problems among the general public in the thick of the global pandemic have also initiated questionnaire development. Questionnaires aimed at assessing how the general population has been affected by the pandemic have also been initiated [17, 18], but these tools are not aimed specifically at current or former COVID-19 patients.

A COVID-19-specific PROM, developed according to internationally recognized guidelines, would ensure high content validity by covering the relevant HRQoL issues for these patients. In addition, a disease specific questionnaire will be more sensitive to changes in patients’ conditions over time, and provide a better ability to capture differences between treatment groups [19].

The objective of this study was to construct an international HRQoL questionnaire for adult patients with COVID-19 according to guidelines [19]. It is intended to be used as an outcome measure in clinical trials and as a descriptive tool at the time of diagnosis, during active disease and treatment, in the recovery phase after the end of isolation, and in principle, for long-term follow-up until full recovery.

## Methods

The development process in this study is based upon the conceptual framework of the European Organisation for Research and Treatment of Cancer (EORTC) Quality of Life Group (QLG). The study follows the international guidelines for questionnaire development from the EORTC QLG, involving four phases (https://qol.eortc.org/manuals/) [19]. Phases I and II of the development process will be presented in this paper. The study team recruited participating countries through the EORTC QLG network and through the WHO COVID-19 clinical management team network. Countries from all continents were approached. Due to time constraints, ethical approvals were required from interested countries within 2 months.

### Phase I generation of HRQoL issues

Three sources were used to compile an exhaustive list of relevant symptoms and HRQoL issues.

### Phase IA: literature review

The study group performed a systematic literature review reported according to PRISMA guidelines [20] to identify all publications containing information on symptoms and other HRQoL issues associated with COVID-19. In the preparation of the data extraction sheet a review of randomly selected publications was performed until no new issues were retrieved (issue saturation after 52 publications). The reviewers pilot-tested the data extraction sheet on 10 abstracts to ensure agreement before the full review of abstracts started. All abstracts were reviewed independently for inclusion by two reviewers, followed by independent data extraction of the included papers. Any disagreements were resolved through discussion and the third reviewer was consulted if needed. Details have been published in PROSPERO (ID = CRD42020185995) [21], and in a separate paper [22]. Issues were included if reported in more than one paper or in one paper with more than 10 patients.

### Phase IB and Phase IC: interview procedure

Native speaking local clinicians and researchers who were members of the (local) research team, performed the interviews. They received detailed information on how to conduct the interviews according to the interview-guide for HCPs in Phase IB (Additional file 1: Appendix 1) and for patients in Phase IC (Additional file 2: Appendix 2). The guides were developed according to the EORTC module development guidelines (https://qol.eortc.org/manuals/) [19]. After written informed consent was received, the interviewer performed the interviews in native language face-to face, via communication platforms (with or without camera), or by telephone. Recording or transcription of the interviews was not compulsory [19].

### Phase 1B: methods, interviews with HCPs

In order to expand or modify the list of issues generated from the systematic review, we included different groups of HCPs. Medical doctors, nurses, researchers, and other relevant HCPs with clinical or research experience with the patients with COVID-19 were eligible. The recruitment followed a pre-specified recruitment matrix (Additional file 1: Appendix 1) which was set up according to guidelines [19]. The interviewer presented the English version of the issue list to the HCP and explained any difficult issues as needed. The HCP rated the relevance of each issue using the categories 1 (not relevant), 2 (a little relevant), 3 (relevant), and 4 (very relevant). For issues with a score of 1–2, they provided reasons. The HCP rated the relative importance by choosing the 15 to 25 issues they would definitely include in a COVID-19 questionnaire, identifying issues they would exclude, and suggesting additional issues they felt were missing. To ensure prioritization of the responses from patients, only issues scored with both very low relevance (mean score < 1.5) and low importance (n < 2) were considered for exclusion after phase IB, while those with borderline relevance and importance were kept in an additional list. Accordingly, phase IB resulted in two issue lists: one main list of issues that the HCP scored as relevant and important, and one additional list of issues with borderline relevance and new issues proposed by the HCP. Differences in the responses from the various HPC groups will be presented in a descriptive manner.

### Phase 1C: methods, interviews with patients

Patients aged 18 years and older, with a previous or current symptomatic verified COVID-19, staying in hospitals, nursing homes, and in private homes during or after the course of the disease were included. Inclusion followed a patient recruitment matrix to ensure a diverse range of participants (Additional file 2: Appendix 2). Patients in intensive care units (ICU) were not eligible, but could be included after discharge from the ICU. Five to 10 patients from each country should be included [19] in Phase IC.

The local partner translated the two lists of issues from phase IB (main and additional list) into native language before the interviews with the patients were conducted. The patients were encouraged to consider all issues they believed to be relevant to the condition and were asked for any missing issues. To reduce the burden of the interview, we had slightly different procedures for the two lists. For the main list, patients were asked to consider each issue; whether they had experienced it, to indicate time of occurrence and duration, and to score its relevance to their own situation using a scale of 1 (not relevant) to 4 (very relevant). To determine relative importance, patients were asked to choose 10 issues they valued particularly highly. For the additional list, patients indicated whether they had experienced the issues and considered them to be important or not. Patients had the opportunity to propose additional issues and were also asked to identify issues that should definitely be included or definitely excluded. Differences in prioritization between patients with early disease (0–4 weeks after diagnosis or up to 14 days after discharge from hospital) and late disease (more than 4 weeks after diagnosis or more than 14 days after discharge from hospital) and duration of symptoms were explored.

### Phase II operationalization

The project group reviewed all issues identified in Phase I. Decisions to exclude issues were based on redundancy (overlap with other closely related issues) and issues that were potentially upsetting or distressing unless found to be important by patients. In addition, if an issue was raised by only one patient and was considered to be of low relevance by the HCP it was considered for exclusion. Issues were retained if experienced by 20 patients or more and scored as important by at least two of them. Issues were excluded if fewer than 20 patients had experienced the issue, unless one or more patients scored it as important. This cut point was chosen to avoid excluding issues that few patients experienced, but still found important. New issues suggested by one or more patients or HCPs were included if the rationale was plausible. Possible overlap between proposed issues was reviewed by the authors and will be psychometrically explored in a later phase.

Each issue was considered in the context of a possible multi-item scale structure, and possible subscales within the questionnaire will be explored in phase III.

The list of relevant issues was operationalised into questions using the system adapted by the EORTC [19]. For item construction, the EORTC Item Library [23] was used to speed up the process of developing and translating appropriate items. It contains an extensive number of validated items (currently more than 900), some of which have been translated into more than 100 languages [24]. All identified issues were searched for, and if available and appropriate, the items were used unchanged. Otherwise, items were constructed. The forward/backward translation followed a modified EORTC translation process [25], with one back translation instead of two, in order to be faster and put less burden on the local partners. This process was supervised and coordinated by the Translation Team Leader in the EORTC Quality of life department (QLD). The response format used was a modified Likert scale with four categories: 1 (not at all), 2 (a little), 3 (quite a bit), and 4 (very much).

The resulting questionnaire was reviewed for clarity of wording and for overlapping questions by the study management team in collaboration with the EORTC QLD by video-conferences and e-mail communication. User representatives were recruited from the established COVID-19 advisory board in Leeds, UK. Nine patients with personal experience with COVID-19 were asked to review the questionnaire (in an advisory capacity rather than as participants) and to give feedback on the wording of instructions and questions and the length of questionnaire. They were also asked to point out if any items were difficult to understand or were confusing or upsetting.

### Statistical analyses

Descriptive statistics are presented as frequencies and percentages for categorical variables and as mean and standard deviation for continuous variables. Missing values are reported as separate categories. Comparison of patients and HCPs are presented descriptively as differences between mean scores to display any large differences in relevance and importance. Statistical testing and subgroup analyses were not performed due to the small number of participants.

## Results

### Phase IA

**Fig. 1:**
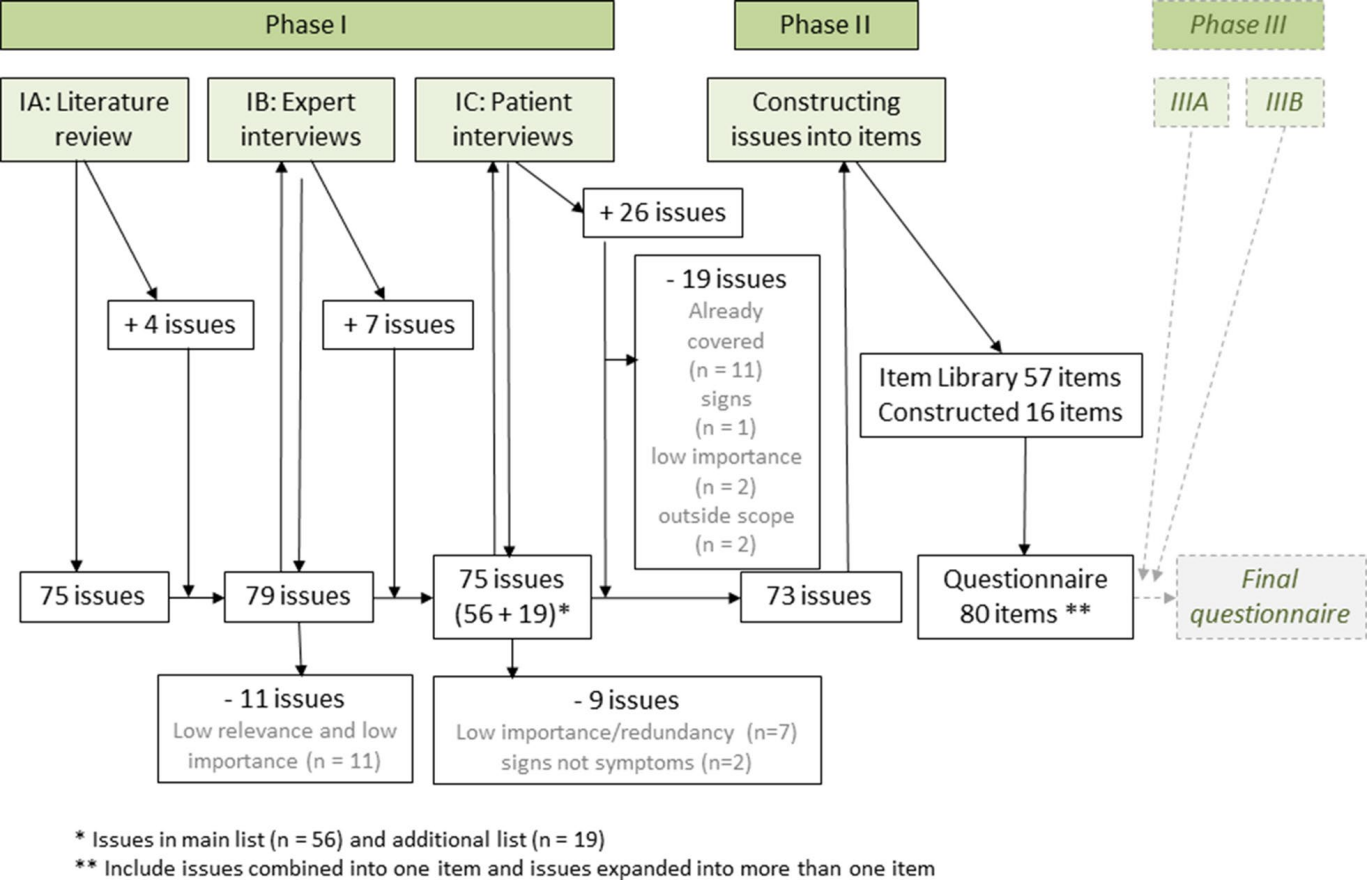
Overview questionnaire development. * Issues in main list (n = 56) and additional list (n = 19). ** Include issues combined into one item and issues expanded into more than one item

In the systematic literature review, 339 of 3342 identified publications were selected for full-text review [22]. A wide range issues reported by patients with COVID-19 at different points in their disease pathway were identified. The final Phase IA-list included 75 issues reported in more than one paper or in one paper with more than 10 patients, and four additional issues considered important to explore further. These four were suicidal thoughts and panic attacks that were briefly mentioned in the literature, and role functioning and overall health and quality of life, included based on existing general questionnaires (Fig. 1).

### Phase IB

**Fig. 2:**
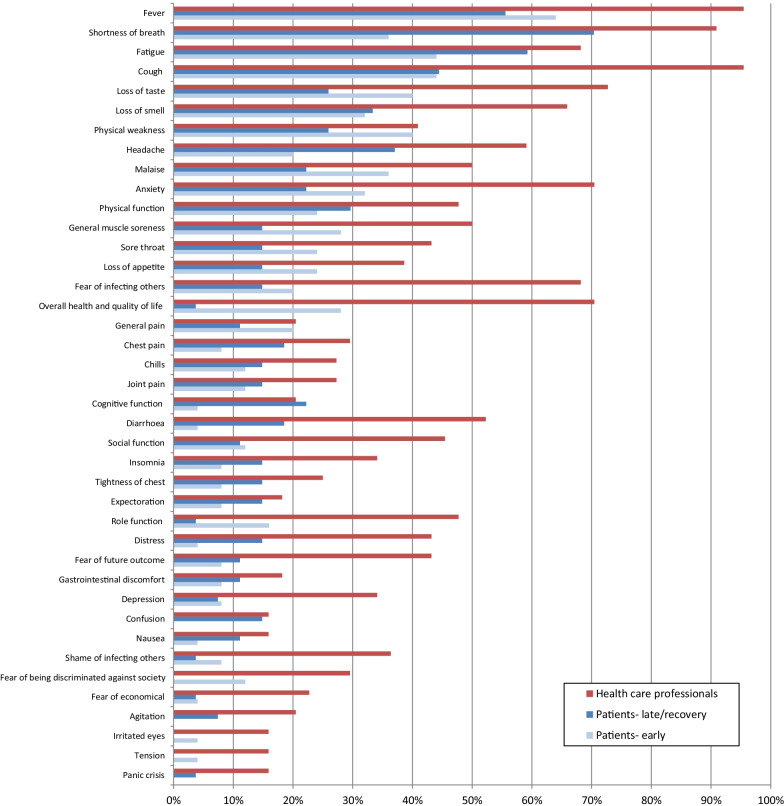
Importance of issues by health care professionals (n = 44) and patients divided by disease status; “early” n = 25 vs. “late/recovery” n = 27. Issues are presented if reported in > 15% of the health care professionals or the patients

In June and July 2020, 44 HCPs with various professional backgrounds from seven countries (Austria, Germany, Italy, Norway, The Philippines, Spain and United Kingdom) were recruited (Table 1). Thirteen HCPs were interviewed face-to-face, nine via video communication, and 22 by telephone. Recording and transcription of the interviews was performed in Germany only. After reviewing the list of 79 issues, their mean relevance scores ranged from 3.8 (cough, shortness of breath) to 1.1 (tinnitus) (Table 2, Additional file 3: Appendix 3). There were no missing values. In general, there were no large differences in the responses between the different HCP groups. For two issues (panic crisis and depression), the differences in the mean relevance scores were 1.0 and 1.1, with the highest score amongst the nurses and lowest amongst the doctors. For one issue (chest discomfort), the difference was 1.0 with the highest score in nurses and lowest in other HCPs. For another 17 issues, the differences were between 0.5 and 1.0, and the nurses had the highest scores in 12 of these. Most HCPs considered fever, cough, shortness of breath, loss of taste, and anxiety as important (Fig. 2). Anxiety, depression and insomnia were prioritized by a lower proportion of doctors compared to the other HCP groups. In total, the doctors and nurses proposed seven new issues: feeling lonely, unable to cope, indifference, worries about being isolated from or abandoned by family and friends, worries about being abandoned by health care personnel, and communication problems with health care personnel.

**Table 1:** Health care professionals characteristics, n = 44

Countries	Medical doctors	Nurses	Other clinical staff	Researchers	Total
Norway	2	2	1	1	6
United Kingdom	3	2	0	1	6
Austria	2	2	1	1	6
Germany	4	2	1	1	8
Spain	2	2	1	1	6
The Philippines	2	2	1	1	6
Italy	3	2	1	0	6
Total	18	14	6	6	44

**Table 2:** Relevance of issues^a^

Issue # in English	Health care professionals N = 44 Relevance (mean score)	Patients N = 52 Relevance (mean score)	Difference between means^b^
Shortness of breath	3.8	3.7	0.1
Cough	3.8	3.1	0.7
Fever	3.7	3.4	0.3
Social function	3.6	3.2	0.4
Overall health and quality of life	3.6	3.0	0.6
Fatigue	3.5	3.4	0.1
Physical function	3.5	3.3	0.2
Distress	3.4	3.1	0.3
Worries about infecting others	3.4	3.0	− 0.4
Role function	3.4	2.9	0.5
Loss of smell	3.3	3.8	− 0.5
Loss of taste	3.3	3.5	− 0.2
Anxiety	3.3	3.4	− 0.1
Headache	3.3	3.2	0.1
Malaise	3.3	3.1	0.2
Worries about future outcome	3.3	3.0	0.3
Physical weakness	3.2	3.1	0.1
General muscle soreness or pain	3.2	3.0	0.2
Shame or guilt of infecting others	3.1	3.0	− 0.1
Diarrhoea	3.0	3.0	0.0
Chest discomfort	2.9	3.4	− 0.5
Tension	2.9	3.4	− 0.5
Loss of appetite	2.9	2.9	0.0
Sore throat	2.9	2.7	0.2
Chest pain	2.8	3.6	− 0.8
Chest congestion	2.8	3.5	− 0.7
Agitation	2.8	3.5	− 0.7
Depression	2.8	3.4	− 0.6
Insomnia	2.8	3.0	− 0.2
Worries about being discriminated against society	2.7	3.1	− 0.4
Cognitive function	2.7	3.1	− 0.4
Joint pain	2.7	2.8	− 0.1
General pain	2.6	3.2	− 0.6
Chills	2.6	2.8	− 0.2
Expectoration	2.5	2.8	− 0.3
Worries about economic difficulties	2.5	2.6	− 0.1
Heart palpitations	2.4	3.2	− 0.8
Panic crisis	2.3	3.0	− 0.7
Nausea	2.3	3.1	− 0.8
Extensive sweating	2.3	2.8	− 0.5
Confusion	2.2	3.3	**− 1.1**
Gastrointestinal discomfort	2.2	3.3	**− 1.1**
Drowsiness	2.2	2.8	− 0.6
Abdominal pain	2.2	2.4	− 0.2
Runny nose	2.2	2.2	0.0
Vomiting	2.1	3.3	**− 1.2**
Nasal congestion	2.1	2.2	− 0.1
Throat congestion	2.0	2.7	− 0.7
Sore eyes	2.0	2.4	− 0.4
Anger	2.0	3.0	**− 1.0**
Back pain	1.9	3.1	**− 1.2**
Sneezing	1.9	2.2	− 0.3
Muscle stiffness	1.8	2.8	−** 1.0**
Dizziness	1.8	2.2	− 0.4
Vision problems	1.5	2.7	**− 1.2**
Hearing loss	1.3	2.3	**− 1.0**

^a^Response categories 1 (not relevant), 2 (a little relevant), 3 (relevant), 4 (very relevant)

^b^Positive values, higher relevance reported by health care professionals. Negative values, higher relevance reported by patients. Values in bold are difference positive or negative ≥ 1

Eleven issues were excluded: nose bleeding, ear pain, uncoordinated movements, seizure, belching, blood in stools, blood in vomit, dysuria, skin pain, urticaria, and tinnitus, because they were identified as candidates for exclusion, and had both low relevance (mean score < 1.3) and low importance (two or fewer regarded issue as important) (Additional file 3: Appendix 3). From phase IB, the main list included 56 issues scored as both relevant and important. The additional list included 19 issues: 12 with borderline relevance (mean score 1.8–1.5) and low importance (three or fewer regarded issues as important) (Additional file 3: Appendix 3), and the seven new issues proposed by the HCPs.

### Phase 1C

In July and August 2020, 52 patients from six countries participated (Table 3). All 75 issues (56 + 19) were experienced by at least two patients (Table 4), and for the 56 main issues, all issues except vomiting and hearing loss were experienced from the time of diagnosis. For 10 of the issues, there were one or two missing values due to patients being unable to recall when the symptom started (Table 4). Psychological symptoms, worries, and reduced functioning lasted the longest (more than five weeks) (Table 4). The mean relevance scores ranged from 3.8 (loss of smell) to 2.2 (dizziness, sneezing, nasal congestion, runny nose) (Table 2). There were no missing values. When comparing the patients’ mean relevance scores with scores from the HCPs, the patients considered back pain, problems with vision, and vomiting more relevant than the HCPs (Table 2). Most patients considered fever, shortness of breath, fatigue, cough, and loss of taste as the five most important issues (Fig. 2). We observed some differences in prioritization of issues between patients with early (acute and sub-acute) disease (n = 25) and patients in recovery (n = 27) (Fig. 2). Loss of taste, physical weakness, malaise, general muscle soreness and overall health and quality of life seemed to be given higher priority by patients with early disease, while shortness of breath, fatigue, headache and diarrhoea were given higher priority by patients in recovery. Confusion and agitation were scored as important among patients in recovery, but not in patients with early disease. Following the patient interviews, nine issues (Fig. 1) were excluded due to (1) low importance or redundancies (n = 7): extensive sweating, runny nose, panic crisis, chest discomfort, suicidal thoughts, abdominal bloating, and fear of cold, and (2) signs not symptoms (n = 2): losing consciousness, and indifference. None of the issues were considered upsetting or distressing. Seven of 26 new issues proposed by the patients were included: dysuria, hair loss, three neuropathic symptoms, dry skin, and dreams and hallucinations. The other 19 issues were not included for the following reasons: covered by issues already included (n = 14), considered to be signs not symptoms (n = 1), scored as not important by the patients (n = 2), and outside the scope of the questionnaire (n = 2).

**Table 3:** Patients characteristics, n = 52

Characteristics	Number (%)
*Age (years)*	
18–40	15 (29)
41–70	26 (50)
≥ 71	11 (21)
*Gender*	
Female	24 (46)
Male	28 (54)
*Country of origin*	
Norway	10 (19)
United Kingdom	8 (15)
Germany	8 (15)
Austria	8 (15)
Spain	10 (19)
The Philippines	8 (15)
*Hospitalisation*	
In hospital	23 (44)
At nursing home	2 (4)
At home	27 (52)
*Disease status*	
Shortly after diagnosis (up to 7 days after diagnosis)	11 (21)
During active disease in institution or at home	9 (17)
Sub-acute (up to 14 days after discharge or four weeks after diagnosis)	5 (10)
Late / recovery (more than 14 days after discharge or four weeks after diagnosis)	27 (52)
*Co-morbidity (Charlson)*	
0–1	40 (77)
≥ 2	12 (23)

**Table 4:** The patients’ experiences of 77 issues (main 56, additional list 19), n = 52

Issue # in English	Have experienced n (%)	Start of experience before diagnosis/during active disease/after end of isolation	Duration of experience (weeks) mean ± SD
*Main list 56 issues*			
Fever	46 (88)	39/7/0	1 ± 1
Fatigue	43 (83)	29/10/4	4 ± 4
Cough	43 (83)	30/10/3	2 ± 3
Physical weakness	40 (77)	20/16/3^a^	4 ± 4
Shortness of breath	36 (69)	16/19/1	3 ± 4
Malaise	35 (67)	22/10/3	4 ± 4
Physical function	34 (65)	15/16/3	6 ± 5
Loss of appetite	31 (60)	18/12/1	3 ± 4
Chills	31 (60)	25/6/0	.6 ± .5
Role function	30 (58)	11/17/2	5 ± 5
General muscle soreness or pain	29 (56)	21/7/1	4 ± 5
Social function	29 (56)	13/15/1	5 ± 5
Worries about infecting others	27 (52)	12/11/4	4 ± 5
Overall health and quality of life	26 (50)	7/14/3^a^	5 ± 5
Extensive sweating	26 (50)	15/9/1^a^	2 ± 2
Anxiety	25 (48)	7/14/4	4 ± 5
Drowsiness	25 (48)	11/12/2	4 ± 4
Diarrhoea	25 (48)	11/14/0	2 ± 4
Loss of taste	24 (46)	8/14/1^a^	3 ± 5
Headache	24 (46)	17/5/2	2 ± 3
Joint pain	23 (44)	14/8/1	3 ± 4
Cognitive function	22 (42)	3/14/5	5 ± 5
Worries about future outcome	22 (42)	3/12/7	5 ± 5
Chest congestion	22 (42)	7/11/3^a^	2 ± 3
Insomnia	22 (42)	4/14/4	3 ± 3
Back pain	21 (40)	13/8/0	2 ± 3
Sore throat	21 (40)	14/7/0	2 ± 3
Dizziness	20 (38)	7/10/3	2 ± 3
Expectoration	20 (38)	8/10/1^a^	3 ± 5
Loss of smell	19 (37)	6/12/0^a^	3 ± 4
General pain	17 (33)	13/2/2	3 ± 4
Chest discomfort	17 (33)	7/6/3^a^	3 ± 3
Distress	16 (31)	4/7/5	4 ± 4
Tension	16 (31)	3/9/4	5 ± 4
Nasal congestion	15 (29)	7/7/1	3 ± 5
Worries about being discriminated against society	14 (27)	1/8/5	6 ± 7
Nausea	14 (27)	6/8/0	1 ± 1
Heart palpitations	13 (25)	4/6/3	3 ± 4
Chest pain	13 (25)	2/8/3	3 ± 4
Sore eyes	13 (25)	4/8/1	4 ± 4
Gastrointestinal discomfort	13 (25)	6/7/0	4 ± 4
Muscle stiffness	12 (23)	6/4/2	2 ± 1
Depression	12 (23)	3/5/3^a^	6 ± 6
Worries about economic difficulties	12 (23)	1/4/7	8 ± 5
Agitation	11 (21)	3/7/1	3 ± 2
Confusion	10 (19)	2/6/2	2 ± 3
Runny nose	10 (19)	3/7/0	2 ± 1
Shame or guilt of infecting others	9 (17)	2/6/1	3 ± 3
Throat congestion	9 (17)	5/4/0	3 ± 4
Sneezing	9 (17)	6/3/0	3 ± 4
Vision problems	9 (17)	2/6/1	3 ± 4
Vomiting	8 (15)	0/8/0	1 ± 1
Abdominal pain	7 (13)	4/3/0	3 ± 5
Anger	6 (12)	2/4/0	4 ± 5
Panic crisis	4 (8)	1/2/1	4 ± 6
Hearing loss	4 (8)	0/2/1^a^	9 ± 5
*Additional list, 19 issues*^*b*^			
Sense of loneliness	15 (29)	na	na
Mucus or extensive amount of saliva in mouth	11 (21)	na	na
Worries about being isolated	10 (19)	na	na
Acid reflux	9 (17)	na	na
Inability to cope	9 (17)	na	na
Red eyes	6 (12)	na	na
Neuropathic pain	6 (12)	na	na
Worries about cold	6 (12)	na	na
Rash	6 (12)	na	na
Worries about being abandoned by family or friends or of being abandoned by professionals or society	6 (12)	na	na
Worries about lack of support from family or friends	5 (10)	na	na
Losing consciousness or fainting	5 (10)	na	na
Constipation	4 (8)	na	na
Indifference	4 (8)	na	
Abdominal distention	4 (8)	na	na
Pruritus	4 (8)	na	na
Hemoptysis	3 (6)	na	na
Difficulties communicating with health personnel wearing personal protective equipment	2 (4)	na	na
Suicidal thoughts	2 (4)	na	na

*SD* standard deviation

^a^Numbers do not add up to 100% due to missing values

^b^Additional list, start and duration of experience not available (na)

### Phase II

Following Phase I, a final list of 73 relevant issues was operationalised into questions (Fig. 1). The study management team, in collaboration with the Translation Team Leader of the EORTC QLD, used the system adapted by the EORTC [24]. From the EORTC Item Library, relevant items were identified for 57 of the issues. Fourteen items needed a slight change in wording; for example, “problems with” was changed to “have you had” to fit the construct of the other items retrieved. For 16 issues, new items were constructed based on the same psychometric principles. The EORTC computerized adaptive test (CAT) database [26] was used to identify suitable items to capture the breadth of the concepts of functioning issues. Cognitive, physical and role functioning were covered by three items each and social functioning by two items. Some issues were combined; skin problem (rash, dry skin and itching) and pain (muscle and joints). Two issues were split into four items (worry about being abandoned by family and health care professionals, and overall health and quality of life). The resulting 80-item provisional questionnaire was reviewed by the study management team and the EORTC QLD. Responses were received from five of the nine people contacted in the COVID-19 advisory board. Two males and three females with previous COVID-19 gave valuable feedback. They found the questionnaire easy to read and covering the breadth of relevant issues. Wording of instructions was slightly changed based on their input. Otherwise, they had no objections to the wording of the questions or length of the questionnaire and none of the items were confusing or upsetting.

## Discussion

This process successfully developed a comprehensive, international, patient-reported COVID-19-specific questionnaire ready to be further validated in the next phase of the project. The questionnaire is intended for adult patients with active disease independent of hospitalization, but not for patients in ICUs. It is also intended for patients in the early and late recovery phase. It can be used in different types of study designs; in randomised clinical trials for comparison of interventions, in longitudinal studies to measure change over time, and in cross-sectional studies to compare different subgroups.

Next, an international population of new COVID-19 patients will validate the questionnaire in phase IIIA and IIIB of the development process, and psychometric properties will be established. In order to capture potential additional symptoms and concerns of patients with long-COVID, interviews with such patients will be performed in phase III. Formal rules for inclusion of possible new issues on long COVID identified in Phase III will be established.

To our knowledge, no other such questionnaire has been developed according to international guidelines. The availability of such a tool is critical to comprehensively assess, in a reliable and valid way, how patients experience this disease at all points in their disease trajectory; to monitor potential treatments and symptom management strategies; and to determine how subgroups of patients may experience the disease differently. It seems likely that COVID-19 will persist at least to some degree, perhaps emerging as outbreaks of mutations, depending on how effective and permanent the immunity provided by vaccines proves to be [27, 28]. A common, exhaustive tool available to all researchers is the key to ensure comparability across future studies on COVID-19 outcomes.

Currently existing generic HRQoL questionnaires such as the SF-36/Rand 36 [29] and EQ-5D [30] are not adequate tools for COVID-19-related HRQoL research as they have not been developed with the aim of capturing all issues specific to these patients. Our literature review and interviews with HCPs and patients showed the considerable breadth of issues for COVID-19 patients, ranging from the more well-known issues such as fever, cough, and shortness of breath, to aspects that are less known such as palpitations and feeling worried about infecting others. Using a generic questionnaire alone would fail to capture all the symptoms and issues that are of importance for these patients and combining them with the COVID-19 specific questionnaire would be preferable.

Nearly all symptoms were experienced by one or more patients from the onset of disease. This emphasizes that clinicians must be aware that COVID-19 infection can have very different clinical features. Currently, there are no specific symptoms that can reliably detect or exclude COVID-19 [31]. Patients rated fatigue as one of the most important issues, and more important in the recovery than in the early phase of the disease. Other studies have also reported fatigue to be common among patients with COVID-19, both during active disease and during recovery [32, 33]. The finding that patients experienced a long duration of psychological symptoms, worries, and reduced functioning emphasizes the importance of including these issues in the questionnaire for it to be able to capture patients’ HRQoL over time. The finding of differences in prioritization of issues between patients with early disease and patients in recovery must be interpreted with caution as the number of participants was small. Dysuria was excluded after the HCP interviews, but re-included after the patient interviews. The discrepancies in ratings between patients and HCPs shows the importance of including patients from the start of the questionnaire development process and prioritizing the responses from patients as recommended [19].

It can be argued that we could have used another strategy such as constructing a COVID-19 symptom checklist, and combining this checklist with established generic instruments into a questionnaire package. However, such a strategy would not have covered specific concerns and difficulties experienced by patients with COVID-19. For instance, “shame or guilt of infecting others”, “worries about being isolated or discriminated against in society” and “difficulties communicating with HCPs using personal protective equipment” would have been missed. Our strategy also confirms the importance of including more than symptoms in the evaluation of HRQoL in such patients.

There are frequent discussions among researchers and clinicians about the length of questionnaires, response burden, and the feasible number of items. However, a review of studies in this field did not find a clear connection between response rates and number of questions [34]. It seems more important to patients that the questions are relevant and easy to understand, as is the case for the current 80-item questionnaire. Previous experiences with the EORTC questionnaires suggest that the use of the EORTC system for constructing items and integrating the same response format used throughout the questionnaire makes it easier and less burdensome for patients to fill out the questionnaire.

One major strength of our study was the collaboration of a multi-language team of researchers with different expertise in the field of quality of life. The involvement of the EORTC QLD Translation Team Leader with linguistic expertise and the use of the EORTC Item Library provided a large number of possible items and translations easily understandable for patients. This was an important starting point for selecting items in an efficient and high quality way. Even though the EORTC Item Library was developed for cancer patients, it describes various symptoms and functional limitations without stating the cause of the problems [24]. Items in this library have already been translated into multiple languages according to international guidelines. If the Item Library did not provide appropriate items, new items were constructed, based upon the same principles as the EORTC items.

The inclusion of patients from different countries, interviewed in different languages, ensured broad coverage of issues and a starting point for cross-cultural consistency. The low frequency of missing values was also a strength, as all participants answered all questions except that a few patients were unable to time when the symptom had started. The methodology used to create the questionnaire is robust and has already produced highly valued quality of life questionnaires for oncology patients [35, 36]. By performing semi-structured interviews at the earliest stage of questionnaire development, content validity is supported, a practice which is also endorsed by FDA Guidance (2007) [37]. In addition, the positive feedback from the review by the patients in the UK COVID-19 advisory board was very reassuring.

The use of a cancer item bank might be considered as a limitation. However, the positive aspects of the utilization of this item bank outweighed the drawbacks, as long as items were modified if needed, and the selected items will thoroughly be tested in patient interviews in the next phase of questionnaire development. The small number of patients in nursing homes and the few patients with considerable comorbidities is a limitation. Possibly, additional symptoms could have been identified with a broader spectrum of patients. Non-European countries had problems prioritizing a methodology study. Therefore, our sample was primarily European, but from the literature review and based on responses from the Filipino patients, symptoms and concerns did not differ substantially between the continents. However, to ensure multicultural adaption, more patients from other continents will be included in the next phase of the development process. When this study started (summer 2020), patients had short-term follow-up. Patients with long-term follow-up will be included in phase III (both with initial interviews—phase IIIA, and in the pretesting in a larger population in phase IIIB), to secure coverage of late effects. It might also be considered a limitation that recording and transcription of interviews were not compulsory in this study. Such recording was not found feasible in the pandemic setting. Many consider recording and transcription to be important for the purpose of reporting accuracy and audit. However, this can also be achieved by the interviewer taking careful field notes at the time of the interview as was done in this study. Common assessment tools are important for monitoring our collective progress in managing the clinical disease and assessing how patients improve over time. The questionnaires potentially available to date to assess COVID-19’s burden were either not specifically created for this disease, centered on a sub-population of COVID-19 patients or on the general public, and with few exceptions have not followed a robust questionnaire development methodology.

In conclusion, this study provides an important message to clinicians to be aware that COVID-19 may occur with a variety of clinical phenotypes. The 80-item COVID-19 specific questionnaire will assess HRQoL issues relevant to adult patients with COVID-19 during active disease and in the recovery phase. The questionnaire will also target patients with long-COVID. It is intended for research independent of design, but may also be relevant for clinical practice. The next steps for the questionnaire resulting from our study are to test patients’ acceptability of its structure and conduct preliminary testing of its psychometric properties. These steps take place in Phases IIIA and IIIB, which are already well underway.

## Supplementary Information

**Additional file 1.** Interview-guide for HCP interviews.

**Additional file 2.** Interview-guide for patient interviews.

**Additional file 3.** Relevance of issues rated by HCPs only.

## Data Availability

Not applicable.
