# Supplementary material for: Health-related quality of life in patients with COVID-19; international development of a patient-reported outcome measure
Source: J Patient Rep Outcomes. 2022 Mar 26;6:26. doi: 10.1186/s41687-022-00434-1 (PMC8962286; doi:10.1186/s41687-022-00434-1)
Supplement: Supplementary file 1 — Additional file 1. Interview-guide for HCP interviews. [file 41687_2022_434_MOESM1_ESM.docx]

**Additional file 1**

**Development of an international questionnaire to assess
patient-reported symptoms related to COVID-19 disease,
the COVID-19 QLQ - ##**

**Phase IB - Interview guide for interviews with Health Care Personnel (HCP)**

**Instruction to interviewer in red**

***Text for interview in cursive***

1. **Preparation for the interview**

General

1. Read through this interview guide before starting the interview
2. Make sure that the six HCP identified in your country covers all the groups in the table below, preferably with a mix of males and females

For each HCP

1. Make sure, if needed in your country, that the HPS has **signed the informed consent before the interview starts**
2. Check that the issue list for the HCP is marked with id.nr and nr. of country
3. Bring or send the issue list to the HCP, make sure that she/he has the list in front of her/him before starting the interview

Open the report sheet on your computer/lap top before starting

1. Confirm that id.nr is the same as for the issue list
2. Fill in type of interview (1 = face-to-face, 2 = communication platform with camera,
   3 = telephone with no camera)
3. Fill in HCP profession, (1 = Medical doctor, 2 = Nurse, 3= other HCP, 4 = Researcher)

**Table 1.**

| **Countries** | **Number** | **Numbering HCP** | **Medical Doctors** | **Nurses** | **Other clinical**  **Staff** | **Researchers** | **Total** |
| --- | --- | --- | --- | --- | --- | --- | --- |
| Norway | 01 | 101-106 | 2 | 2 | 1 | 1 | 6 |
| UK | 02 | 201-206 | 2 | 2 | 1 | 1 | 6 |
| Austria | 03 | 301-306 | 2 | 2 | 1 | 1 | 6 |
| Germany | 04 | 401- 406 | 2 | 2 | 1 | 1 | 6 |
| Spain | 05 | 501-506 | 2 | 2 | 1 | 1 | 6 |
| Croatia | 06 | 601- 606 | 2 | 2 | 1 | 1 | 6 |
| Philippines | 07 | 701-706 | 2 | 2 | 1 | 1 | 6 |

1. **Start of interview with explaining the purpose**

**Opening question** *We are asking for your help in developing a questionnaire that will be used to assess quality of life issues including symptom, functions and concerns of adult patients who have or have had COVID-19 disease. This is an international initiative, involving 7 countries in the first phase, and many more in the next phase where a preliminary questionnaire will be tested in Europe, Asia, Africa and probably USA.*

- **(In countries with informed consent from HPC:** *As you have seen in the consent letter,)
  I will ask you to review a list of issues retrieved trough a thorough literature review of COVID-19 papers reporting symptoms, reduced functions and concerns of these patients*
- *You will be asked to indicate which of these symptoms your patients have experienced and which ones you consider relevant to include in a questionnaire.*
- *Following this, you will be asked to mark those issues you find most important and whether there are any issues you think should be excluded.*
- *I will fill in your answers in the response sheet as we go along. No audio or video recording will be used.*

Place list with issues before the HCP or ask her/him to look at the list she/he has received if using video meeting.

1. **Relevance**

Ask about relevance
Read out each issue on the list and note the HCP’s answer in the report sheet (Excel), column C, response categories 1, 2, 3 or 4

- *Please, look at the list of issues; these are symptoms, functions and concerns that are reported to be relevant to patients with COVID-19.*
- *I will now ask you for each of these issues separately, the extent to which you find it relevant for this patient group using the categories
  1 (not relevant), 2 (a little relevant) 3 (relevant) 4 (very relevant).*

Ask the HCP to comment on the issues she/he found to be **no (1) or little (2) relevance**.
Make notes of the **reasons** in report sheet (Excel), column D

- *Please tell me for each issue where you put 1 (not relevant) or 2 (a little relevant)
  why do you consider it not or only a little relevant?*

1. **Relative importance**

Ask questions on relative importance of issues.
Note the HCP’s answer in the report sheet (Excel) column F with a 1

- *The list of issues is too long to be administered to patients. Please could you mark those 15 - 25 issues that, in your opinion, we should definitely include in the final questionnaire?*

Ask about exclusion.
Make a note of which issues to exclude in the report sheet (Excel) column G with a 1, and the reason in column H.

- *If there are issues that you think should definitely be excluded please mark these also.*
- *For issues that you think should be excluded, could you please tell me the reason?*

1. **Breadth of coverage**

Ask about other relevant issues and write them in the report sheet (Excel), at the bottom of the issue list after the pre-specified issues. In addition, make notes of the reasons in report sheet (Excel), column C

- *Please consider COVID-19 patients at all stages of disease and patients undergoing any type of treatment for this condition. Can you think of anything else that may be relevant to this patient group?*
- *If yes: Please name each of these issues so I can write them down.*

For each additional issue:

- *Could you tell me about this?*

**F. Closing remarks**

Ask the HCP whether she/he has any closing remarks and thank her/him for their effort. Make notes of general comments from the HCP in the assigned box in the Excel sheet.

- *Do you have anything else that you want to add before we close this interview?*
- *Thank you for your time and effort. Would you like to be informed of the results of this project*?

Please provide **your general comments** in the assigned box

Very important: Please review that all tasks have been performed and reported in the Excel sheet

If you have questions, after reading this document and the protocol, do not hesitate to ask me (do not copy all participants).

Attachments:

1. Issue list for HCP
2. Excel report sheet (one Excel file per country, one sheet per interview).
3. Final protocol
4. Template in English, HCP consent form
