# Supplementary material for: Health-related quality of life in patients with COVID-19; international development of a patient-reported outcome measure
Source: J Patient Rep Outcomes. 2022 Mar 26;6:26. doi: 10.1186/s41687-022-00434-1 (PMC8962286; doi:10.1186/s41687-022-00434-1)
Supplement: Supplementary file 2 — Additional file 2. Interview-guide for patient interviews. [file 41687_2022_434_MOESM2_ESM.doc]

**Additional file 2**

**Development of an international questionnaire to assess
patient-reported symptoms related to COVID-19 disease,
the COVID-19 QLQ - ##**

**Phase IC - Interviews with Patients**

**Instruction to interviewer in red**

***Text for interview in cursive***

1. **Preparation for the interview**

Please use the allocated country number and patient numbers for identification

| **Countries** | **Number** | **Numbering Patients** |
| --- | --- | --- |
| Norway | 01 | 101-108 |
| UK | 02 | 201-208 |
| Austria | 03 | 301-308 |
| Germany | 04 | 401-408 |
| Spain | 05 | 501-508 |
| Croatia | 06 | 601-608 |
| Philippines | 07 | 701-708 |

**General**

Make sure that the eight patients identified in your country covers all the groups in the table below.
Remember that one patient will cover many boxes

|  |  | **Country** | | | | | | | |
| --- | --- | --- | --- | --- | --- | --- | --- | --- | --- |
|  |  | **Norway** | **UK** | **Germany** | **Austria** | **Spain** | **Croatia** | **the Philippines** | **Total** |
| **Number of patients** | **Total 56** | **8** | **8** | **8** | **8** | **8** | **8** | **8** | **Total 100%** |
| **Age (years)** |  |  | | | | | | | |
| 18-40 | ≥ 2 | ! |  |  |  |  |  |  | ≥ 20 |
| 41-70 | ≥ 2 | x |  |  |  |  |  |  | ≥ 20 |
| ≥ 71 | ≥ 2 | # |  |  |  |  |  |  | ≥ 20 |
| **Gender** |  |  |  |  |  |  |  |  |  |
| Female | ≥ 3 | ! x |  |  |  |  |  |  | ≥ 30 |
| Male | ≥ 3 | # |  |  |  |  |  |  | ≥ 30 |
| **Hospitalisation** |  |  | | | | | | | |
| In hospital | ≥ 2 | #x |  |  |  |  |  |  | ≥ 20 |
| At nursing home | ≥ 2 |  |  |  |  |  |  |  | ≥ 20 |
| At home | ≥ 2 | ! |  |  |  |  |  |  | ≥ 20 |
| **Disease status** |  |  | | | | | | | |
| shortly after diagnosis (up to 7 days after diagnosis) | ≥ 1 | x |  |  |  |  |  |  | ≥ 20 |
| during active disease in institution or at home | ≥ 1 |  |  |  |  |  |  |  | ≥ 20 |
| subacute (up to 14 days after discharge or four weeks after diagnosis) | ≥ 1 | ! |  |  |  |  |  |  | ≥ 20 |
| Late / recovery (more than 14 days after discharge or four weeks after diagnosis) | ≥ 1 | # |  |  |  |  |  |  | ≥ 20 |
| **Co-morbidity (Charlson)** |  |  | | | | | | | |
| 0-1 | ≥ 2 | ! x |  |  |  |  |  |  | ≥ 30 |
| > 2 | ≥ 2 | # |  |  |  |  |  |  | ≥ 30 |

Example on how to fill in the Matrix:

- # Male, 85 years old, cardiovascular disease and diabetes, treated in hospital more than four weeks ago
- ! Female, 35 years old, no comorbidities, treated at home, diagnosed two weeks age
- X Female, 45 years old, diabetes, in hospital, first week of disease

**Follow-up questions**

The use of follow-up questions or “probes” will be required in the majority of interviews.

**If the answer is too general and indefinite, the follow-up may be**

- *In what way?*
- *Just how do you mean?*
- *Can you give me an example?*

**If the answer is incomplete, the questions may be:**

- *Any other reasons?*
- *Would you tell me a little more about that?*

**Other suggestions:**

- *What makes you think this?*
- *What was there about the issue that made you feel that way?*

**For each Patient**

1. Make sure, that the patient has signed the informed consent before the interview starts.
2. Bring a copy of the two issue lists
   1. Main list for interviews,
   2. Supplementary list)
3. Fill in the CRF with patient characteristic

| **Country** |  |
| --- | --- |
| **Language** |  |
| **Collaborating centre** |  |
| **Name of contact person** |  |
| **Patient local study number** |  |
| **Introduction**   - *We are asking for your help in making a questionnaire which will be used to assess the experiences of patients who have or have had COVID-19 disease.* - *I would like to ask you a few things about your health.  Can you tell me about your experiences with the disease in general?* | |
|  | |
| - *What was the most stressful experience for you* | |
|  | |
| - *Can you tell me about your initial symptoms?* | |
|  | |
| **Issue list (forwarded to patients beforehand)**  **Part IA**  *Here you see a list of patient* ***experiences*** *related to the COVID-19 disease that have been reported in the literature or by health care professionals*   - *We will now go through the list and, for each one, I will ask you to tell me*   - *the* ***extent to which you have experienced*** *it during your illness, and,*   - *the* ***relevance*** *of this issue, independent of your experience.* - *For each of the issues you have experienced, could you please indicate*   - ***the start*** *(before diagnosis, during active disease, after end of isolation) and*   - ***the duration*** *of this issue (in days/weeks)?* | |

| **Part II**  **Relative importance** (after indicating additional issues) **– prioritized issues**   - *I would like to ask you to indicate 10 of these problems,* ***including the additional problems you have mentioned*** *yourself that you find most important and/or most stressful*   **Redundancy, exclusion of issues – comment box**   - *Is it any of the issues that you find to be overlapping or to have the same meaning as others, and therefore, should be taken out?* - *Are there any of the issues that you find upsetting or that you think should not be included****?*** |
| --- |
|  |

|  | | | **Part IA** | | | | **Part II** | |
| --- | --- | --- | --- | --- | --- | --- | --- | --- |
| **Nr** | **Issue #**  **in local language** | **Issue #**  **in English** | **Have experienced?**  **No = 0**  **Yes = 1** | **If yes,**  **Start of experience:**  **1 = before diagnosis**  **2 = during active disease**  **3 = after end of isolation** | **If yes, duration**  **In days or weeks** | **Relevance**  **1 = not relevant**  **2 = a little relevant**  **3 = quite relevant**  **4 = very relevant** | **Prioritized** | **Comments** |
| 1 |  | Fever |  |  |  |  |  |  |
| 2 |  | Chills/shivering |  |  |  |  |  |  |
| 3 |  | Fatigue/Asthenia |  |  |  |  |  |  |
| 4 |  | Malaise/ feeling unwell |  |  |  |  |  |  |
| 5 |  | Physical weakness |  |  |  |  |  |  |
| 6 |  | Extensive sweating/ night sweats |  |  |  |  |  |  |
| 7 |  | Dizziness |  |  |  |  |  |  |
| 8 |  | Drowsiness |  |  |  |  |  |  |
| 9 |  | Confusion |  |  |  |  |  |  |
| 10 |  | Cough |  |  |  |  |  |  |
| 11 |  | Shortness of breath/ Respiratory distress |  |  |  |  |  |  |
| 12 |  | Expectoration/Phlegm production |  |  |  |  |  |  |
| 13 |  | Sore throat |  |  |  |  |  |  |
| 14 |  | Stuffed throat/  throat congestion |  |  |  |  |  |  |
| 15 |  | Sneezing |  |  |  |  |  |  |
| 16 |  | Mucus nose/Nasal congestion |  |  |  |  |  |  |
| 17 |  | Runny nose/coryza |  |  |  |  |  |  |
| 18 |  | Hearing loss |  |  |  |  |  |  |
| 19 |  | Irritated/sore Eyes |  |  |  |  |  |  |
| 20 |  | General Pain |  |  |  |  |  |  |
| 21 |  | General muscle soreness/pain |  |  |  |  |  |  |
| 22 |  | Headache |  |  |  |  |  |  |
| 23 |  | joint pain |  |  |  |  |  |  |
| 24 |  | Back pain |  |  |  |  |  |  |
| 25 |  | Rigor/muscle stiffness |  |  |  |  |  |  |
| 26 |  | Loss Of Taste |  |  |  |  |  |  |
| 27 |  | Loss Of Smell |  |  |  |  |  |  |
| 28 |  | Sight/vison problems |  |  |  |  |  |  |
| 29 |  | Diarrhoea |  |  |  |  |  |  |
| 30 |  | Nausea |  |  |  |  |  |  |
| 31 |  | Vomiting |  |  |  |  |  |  |
| 32 |  | Loss of appetite |  |  |  |  |  |  |
| 33 |  | Stomachache/Abdominal pain |  |  |  |  |  |  |
| 34 |  | Gastrointestinal discomfort (different from pain) |  |  |  |  |  |  |
| 35 |  | Rash | Moved to additional list |  |  |  |  |  |
| 36 |  | Anxiety |  |  |  |  |  |  |
| 37 |  | Depression |  |  |  |  |  |  |
| 38 |  | Distress |  |  |  |  |  |  |
| 39 |  | Tension |  |  |  |  |  |  |
| 40 |  | Agitation |  |  |  |  |  |  |
| 41 |  | Anger |  |  |  |  |  |  |
| 42 |  | Iinsomnia/ difficult falling or staying asleep |  |  |  |  |  |  |
| 43 |  | Panic crisis |  |  |  |  |  |  |
| 44 |  | Suicidal thoughts | Moved to additional list |  |  |  |  |  |
| 45 |  | Chest Pain |  |  |  |  |  |  |
| 46 |  | Tightness Of Chest/ Chest congestion |  |  |  |  |  |  |
| 47 |  | Chest discomfort |  |  |  |  |  |  |
| 48 |  | Heart Palpitations |  |  |  |  |  |  |
| 49 |  | Fear Of/ concerns for/ worries about Infecting Others |  |  |  |  |  |  |
| 50 |  | Shame/ guilt of infecting others |  |  |  |  |  |  |
| 51 |  | Fear Of/ concerns for/ worries about being discriminated against society |  |  |  |  |  |  |
| 52 |  | Fear Of/ concerns for/ worries about future outcome |  |  |  |  |  |  |
| 53 |  | Fear Of/ concerns for/ worries about Economical |  |  |  |  |  |  |
| 54 |  | Physical function (able to walk, carry out strenious activities, activity of daily living) |  |  |  |  |  |  |
| 55 |  | Role function (able to carry out ordinary work/daily activities and/or hobbies/ leisure time activities) |  |  |  |  |  |  |
| 56 |  | Cognitive function (Concentration and memory) |  |  |  |  |  |  |
| 57 |  | Social function (interferrence with family or social life) |  |  |  |  |  |  |
| 58 |  | Overall health and quality of life –  taking everythning in consideration, how is your health and quality of life… |  |  |  |  |  |  |

**Part IB**

*Can you think of anything else that you have experienced/had to cope with during your illness that we have not already talked about and is not included in this list?*

|  | | | **Part IB** | | | **Part II** | |
| --- | --- | --- | --- | --- | --- | --- | --- |
| **Nr** | **Additional issue # in local language** | **Additional issue # in English** | **Have experienced?**  **No = 0**  **Yes = 1** | **Start and duration** | **Relevance**  **1 = not relevant**  **2 = a little relevant**  **3 = quite relevant**  **4 = very relevant** | **Prioritized** | **Comments** |
|  |  |  |  |  |  |  |  |
|  |  |  |  |  |  |  |  |
|  |  |  |  |  |  |  |  |
|  |  |  |  |  |  |  |  |
|  |  |  |  |  |  |  |  |
|  |  |  |  |  |  |  |  |

**Part III**

| **Additional list** | | | | |
| --- | --- | --- | --- | --- |
| - *Here is another list of issues mentioned in literature. Have you experienced any of these issues?* - *Do you find any of them to be particularly important?* - *Some patients experienced what is called* ***silent hypoxemia*** *(sudden change from no apparent symptoms to breathing problems). Did you experience this? If yes, could you please describe the initial symptoms before the health care personnel realized that you had problems?* | | | | |
| **Nr** | **Additional issue # in local language** | **Additional issue # in English** | **Have experienced?**  **No = 0**  **Yes = 1** | **Find particularly important** |
| 59 |  | Losing consciousness or fainting |  |  |
| 60 |  | Hemoptysis/blood in the phlegm |  |  |
| 61 |  | Mucus/saliva in mouth; change in amount and/or consistency |  |  |
| 62 |  | Ear pain | Excluded |  |
| 63 |  | Red Eyes |  |  |
| 64 |  | Neurological/neuropatic Pain, nerve pain |  |  |
| 65 |  | Seizure/epileptic fits | Excluded |  |
| 66 |  | Constipation |  |  |
| 67 |  | Acid Reflux/heartburn |  |  |
| 68 |  | Abdominal distention/bloating |  |  |
| 69 |  | Itching/ pruritus |  |  |
| 70 |  | Fear Of/ concerns for/ worries about cold |  |  |
| 71 |  | Fear Of/ concerns for/ worries about lack of support from family or friends |  |  |
| 72 |  | Fear Of/ concerns for/ worries about being isolated? |  |  |
| 73 |  | Sense of loneliness |  |  |
| 74 |  | Inability to cope |  |  |
| 75 |  | Fear Of/ concerns for/ worries about being abandoned by family or friends + Fear of being abandoned by professionals or society |  |  |
| 76 |  | Indifference |  |  |
| 77 |  | Communication issues, difficult to communicate with health personnel wearing personal protective equipment (PPE) |  |  |
| 35 |  | Rash |  |  |
| 44 |  | Suicidal thoughts |  |  |
| Comments from patient | | | | |
| Comments from interviewer | | | | |
