# Supplementary material for: Health-related quality of life in patients with COVID-19; international development of a patient-reported outcome measure
Source: J Patient Rep Outcomes. 2022 Mar 26;6:26. doi: 10.1186/s41687-022-00434-1 (PMC8962286; doi:10.1186/s41687-022-00434-1)
Supplement: Supplementary file 3 — Additional file 3. Relevance of issues rated by HCPs only. [file 41687_2022_434_MOESM3_ESM.docx]

**Additional file 3**

**Appendix 3. Relevance of issues rated by health care professionals (HCPs) only**

| **Issue #**  **in English** | Relevance  mean score* |
| --- | --- |
| **The 12 issues included in additional list phase IC** |  |
| Mucus or extensive amount of saliva in mouth | 1.8 |
| Red eyes | 1.8 |
| Rash | 1.8 |
| Suicidal thoughts | 1.8 |
| Haemoptysis | 1.7 |
| Losing consciousness or fainting | 1.6 |
| Neuropathic pain | 1.6 |
| Worries about cold | 1.6 |
| Constipation | 1.5 |
| Acid reflux | 1.5 |
| Abdominal distention | 1.5 |
| Pruritus | 1.5 |
| **The 11 issues excluded following the HCPs interviews** |  |
| Nose bleeding (epistaxis) | 1.3 |
| Ear pain | 1.3 |
| Uncoordinated movements | 1.3 |
| Seizure | 1.3 |
| Belching | 1.3 |
| Blood in stools (melena) | 1.3 |
| Blood in vomit (hematemesis) | 1.3 |
| Dysuria | 1.3 |
| Skin pain | 1.3 |
| Urticaria (hives) | 1.3 |
| Tinnitus | 1.1 |

*Response categories 1 (not relevant), 2 (a little relevant), 3 (relevant), 4 (very
